# Supplementary material for: The changing impact of rural electrification on Indian agriculture
Source: Nat Commun. 2023 Oct 25;14:6780. doi: 10.1038/s41467-023-42533-7 (PMC10600167; doi:10.1038/s41467-023-42533-7)
Supplement: Supplementary file 1 — Supplementary Information [file 41467_2023_42533_MOESM1_ESM.pdf]

The Changing Impact of Rural Electrification on Indian Agriculture

Sudatta Ray[1,2,*] and Hemant K Pullabhotla[3]

[1]Department of Geography, National University of Singapore, Singapore

[2]Environmental Studies, Division of Social Science, Yale-NUS College, Singapore, sray@nus.edu.sg

[3]Department of Economics, Deakin Business School, Australia, h.pullabhotla@deakin.edu.au

[*]Corresponding author

Supplementary Information

Supplementary Notes

1. A 10 horsepower pump is capable of delivering a flow rate of 0.01m/s at a depth of 70m assuming a high motor efficiency of 90%. More realistic assumptions of pump efficiency will require greater pump capacity to deliver similar flow rates.

2. Figure 2 uses icons from noun project, which are open sourced and under creative commons license.

Supplementary Figures

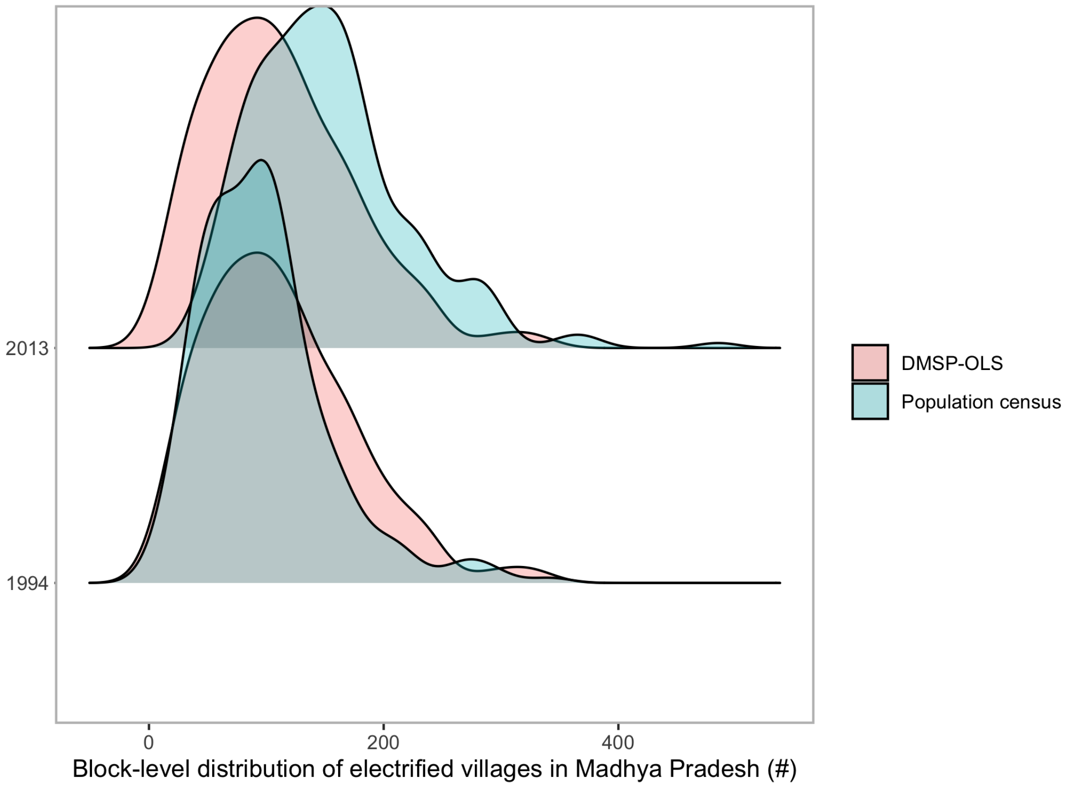

Supplementary Figure 1: Density distribution of the number of electrified villages in a block in Madhya Pradesh as defined by the Defense Meteorological Program Operation Line Scan System (DMSP-OLS) night time luminosity data, and population census (Asher et al., 2021; Census of India, 1991, 2011). A village is classified electrified if any pixel in that village registered a nightlight luminosity greater than 0.

Supplementary Tables

Supplementary Table 1: Block-level association between late electrification and energy source for groundwater pumps in Madhya Pradesh

	Groundwater wells		
	Total	With electric pumps	With diesel pumps
Log nightlights	1,484.0***	1,503.9***	-7.156
	(296.9)	(287.0)	(35.79)
Log nightlights × *PC*	-974.4**	-1,001.0**	-33.31
	(456.4)	(424.1)	(64.09)
Total villages	-0.4793	-0.4824	-0.2428
	(2.287)	(2.074)	(0.5760)
Fixed-effects			
District	Yes	Yes	Yes
Year	Yes	Yes	Yes
Fit statistics			
Observations	699	699	699
R^2	0.71954	0.72575	0.43254
Within R^2	0.11919	0.12941	0.00203

Note: *p<0.1; **p<0.05; ***p<0.01

Standard errors are clustered at the district level.

Linear regression of the number of groundwater wells with pumps on the number of electrified households estimated using equation 1 and by matching minor irrigation census and SHRUG data, see methods. Asher et al. (2021). Policy Change *PC* is a binary indicator for districts that were majorly electrified after policy change (methods). Nightlights refers to total light luminosity values which ranges from 0 to 63 and are calibrated by Elvidge et al. (2014) for consistent measure across the range of years between 1994-2013.

Supplementary Table 2: Association between electrification quantiles and and energy source for groundwater pumps

	Groundwater wells		
	Total	With electric pumps	With diesel pumps
Electrified households	0.0711	0.3161***	-0.1130***
	(0.0802)	(0.0445)	(0.0207)
Electrified households × 15th electrification quantile	-0.1146	-0.3047***	0.1063***
	(0.0775)	(0.0467)	(0.0283)
Electrified households × 25th electrification quantile	-0.0496	-0.2793***	0.1333***
	(0.0828)	(0.0478)	(0.0235)
Electrified households × 50th electrification quantile	0.0203	-0.1738***	0.0832***
	(0.0811)	(0.0470)	(0.0187)
Electrified households × 75th electrification quantile	0.0211	-0.1208**	0.0457**
	(0.0895)	(0.0554)	(0.0187)
Total Households	0.0324**	-0.0100	0.0029
	(0.0140)	(0.0066)	(0.0110)
Fixed-effects			
District	Yes	Yes	Yes
year	Yes	Yes	Yes
Fit statistics			
Observations	969	969	969
R^2	0.72284	0.91002	0.80176
Within R^2	0.06949	0.55825	0.13479

Note: *p<0.1; **p<0.05; ***p<0.01

Standard errors are clustered at the district level.

Linear regression of the number of groundwater wells with pumps on the number of electrified households estimated using equation 1 and panel dataset constructed from district-level population and minor irrigation census data. Electrification quantiles are based on 2001 electrification rates in all districts. The highest electrification quantile 85th percentile has been left out.

Supplementary Table 3: Association between electrification quantiles and expansion in annual and season-wise area irrigated

	Irrigated area (ha)		
	Annual	*Kharif*	*Rabi*
Electrified households	0.3509**	0.2091*	0.2714***
	(0.1636)	(0.1127)	(0.0909)
Electrified households × 15th electrification quantile	-0.3442*	-0.1590	-0.2680***
	(0.1762)	(0.1162)	(0.0999)
Electrified households × 25th electrification quantile	-0.4258***	-0.2104*	-0.2940***
	(0.1609)	(0.1128)	(0.0926)
Electrified households × 50th electrification quantile	-0.2474	-0.1255	-0.2065**
	(0.1596)	(0.1121)	(0.0912)
Electrified households × 75th electrification quantile	-0.0730	-0.0174	-0.1379
	(0.1716)	(0.1196)	(0.0968)
Total households	0.0613	0.0141	-0.0197
	(0.0451)	(0.0188)	(0.0209)
Cumulative monthly rain†	-1.953	-7.954**	29.02
	(6.303)	(3.197)	(27.31)
Fixed-effects			
District	Yes	Yes	Yes
Year	Yes	Yes	Yes
Fit statistics			
Observations	969	969	969
R^2	0.86501	0.85675	0.85491
Within R^2	0.34513	0.33112	0.25470

Note: *p<0.1; **p<0.05; ***p<0.01

Standard errors are clustered at the district level.

Linear regression of the area irrigated by groundwater wells across seasons on the number of electrified households estimated using equation 1 and panel dataset constructed from district-level population and minor irrigation census data. Electrification quantiles are based on 2001 electrification rates in all districts. The highest electrification quantile 85th percentile has been left out.

†Annual, average rainfall during November to March and average rainfall during June to October were used for annual, *Rabi* and *Kharif* cultivation seasons respectively.

Supplementary Table 4: Association between late-electrification and expansion in annual and season-wise area irrigated without controlling for seasonal rainfall

	Irrigated area (ha)					
	Annual (ha)		*Kharif (ha)*		*Rabi (ha)*	
	(1)	(2)	(3)	(4)	(5)	(6)
Electrified households	0.1396***	-0.0420	0.1027***	-0.0065	0.0789***	0.0029
	(0.0494)	(0.0427)	(0.0250)	(0.0218)	(0.0204)	(0.0204)
	(0.0606)	(0.0528)	(0.0329)	(0.0263)	(0.0289)	(0.0263)
Electrified Households × *PC*	-0.2070***	-0.0503	-0.0983***	-0.0041	-0.1140***	-0.0484*
	(0.0606)	(0.0528)	(0.0329)	(0.0263)	(0.0289)	(0.0263)
Wells with electric pumps		1.150***		0.6916***		0.4814***
		(0.1593)		(0.1101)		(0.0728)
Total households	0.0636	0.0781*	0.0185	0.0272	-0.0193	-0.0133
	(0.0435)	(0.0434)	(0.0184)	(0.0182)	(0.0192)	(0.0194)
Fixed-effects						
District	Yes	Yes	Yes	Yes	Yes	Yes
Year	Yes	Yes	Yes	Yes	Yes	Yes
Fit statistics						
Observations	969	969	969	969	969	969
R^2	0.86323	0.87402	0.85313	0.87127	0.85122	0.85969
Within R^2	0.33650	0.38883	0.31426	0.39892	0.23573	0.27922

Note: *p<0.1; **p<0.05; ***p<0.01

Standard errors are clustered at the district level.

Linear regression of the area irrigated by groundwater wells across seasons on the number of electrified households estimated using equation 1 without rainfall controls and panel dataset constructed from district-level population and minor irrigation census data. Policy Change (*PC*) is a binary indicator for districts that were majorly electrified after policy change (methods).
†Annual, average rainfall during November to March and average rainfall during June to October were used for annual, *Rabi* and *Kharif* cultivation seasons respectively.

Supplementary Table 5: Mean monthly per capita consumption across percentiles

Consumption Percentile	PC districts	non-PC districts	p Value
15th Consumption Percentile	544.67	550.25	0.37
25th Consumption Percentile	745.57	745.33	0.93
50th Consumption Percentile	955.98	948.42	0.02
75th Consumption Percentile	1268.68	1273.48	0.35
85th Consumption Percentile	2568.37	2504.39	0.70

Two-sided t-tests of mean monthly per capita consumption between Policy Change (PC) and non-Policy Change (non-PC) districts using nationally representative survey data of agricultural households in 2012-13 (methods). PC is a binary indicator for districts that were majorly electrified after policy change (methods). Consumption percentiles are constructed using all agricultural households across both PC and non-PC districts.

Supplementary Table 6: Differences in agricultural land irrigated by households in non-Policy Change (non-PC) and Policy Change (PC) districts during *Rabi* and *Kharif* during 2012-13

	Irrigated area during (ha)	
	Rabi	Kharif
15th Consumption Percentile × *PC*	0.04	-0.20^{***}
	(0.05)	(0.08)
25th Consumption Percentile × *PC*	0.16	0.37^{***}
	(0.10)	(0.14)
50th Consumption Percentile × *PC*	-0.08^{**}	0.03
	(0.04)	(0.07)
75th Consumption Percentile × *PC*	−0.08	0.05
	(0.05)	(0.08)
85th Consumption Percentile × *PC*	−0.08	0.02
	(0.05)	(0.08)
25th Consumption Percentile	−0.09	-0.52^{***}
	(0.11)	(0.16)
50th Consumption Percentile	0.16^{***}	-0.16^{*}
	(0.05)	(0.09)
75th Consumption Percentile	0.16^{***}	−0.15
	(0.06)	(0.10)
85th Consumption Percentile	0.18^{***}	0.01
	(0.06)	(0.10)
Cultivated Area	0.77^{***}	0.46^{***}
	(0.04)	(0.08)
Awareness about rice MSP	−0.01	0.19^{***}
	(0.02)	(0.03)
Awareness about wheat MSP	0.12^{***}	−0.0003
	(0.02)	(0.04)
Land Owned	−0.01	−0.02
	(0.01)	(0.05)
Household size	0.01^{***}	0.03^{***}
	(0.003)	(0.01)
Middle school or higher education	−0.01	0.02
	(0.01)	(0.02)
Received agricultural training	−0.07	0.004
	(0.04)	(0.05)
Non-farm expenses	−0.0000	−0.0000
	(0.0000)	(0.0000)
Non-farm value	0.0000	0.0000
	(0.0000)	(0.0000)
Cumulative monthly rain †	−0.0000	0.0000
	(0.0000)	(0.0000)
Constant	-0.25^{***}	0.10
	(0.07)	(0.11)
State Controls?	Yes	Yes
Observations	11,182	11,182
Adjusted R^2	0.84	0.56

Note: *p<0.1; **p<0.05; ***p<0.01

Standard errors are clustered at the district level.

Linear regression of groundwater irrigated areas across seasons on consumption percentiles of households estimated using equation 2 and nationally representative survey data of agricultural households in 2012-13, see Data. Policy Change *PC* is a binary indicator for districts that were majorly electrified after policy change (methods). Consumption percentiles are constructed using all agricultural households across both PC and non-PC districts. The lowest consumption percentile (15th) in non-Policy Change (non-PC) district has been left out.

†Average rainfall during November to March and average rainfall during June to October were used for *Rabi* and *Kharif* cultivation seasons respectively.

Supplementary Table 7: Differences in agricultural land irrigated by households in non-Policy Change (non-PC) and Policy Change (PC) districts using alternate specifications for mean monthly per capita consumption

	Rabi Irrigated area (ha)		
	Full	Specification #2†	Specification #3†
15th Consumption Percentile × PC	0.04	0.05	0.01
	(0.05)	(0.05)	(0.05)
25th Consumption Percentile × PC	0.16	0.10	−0.001
	(0.10)	(0.08)	(0.07)
50th Consumption Percentile × PC	-0.08^{**}	-0.08^{*}	0.07
	(0.04)	(0.04)	(0.06)
75th Consumption Percentile × PC	−0.08	−0.09	-0.14^{***}
	(0.05)	(0.06)	(0.04)
85th Consumption Percentile × PC	−0.08	-0.09^{*}	−0.07
	(0.05)	(0.05)	(0.05)
25th Consumption Percentile	−0.09	−0.02	−0.001
	(0.11)	(0.09)	(0.08)
50th Consumption Percentile	0.16^{***}	0.17^{***}	−0.04
	(0.05)	(0.06)	(0.07)
75th Consumption Percentile	0.16^{***}	0.18^{***}	0.15^{***}
	(0.06)	(0.07)	(0.05)
85th Consumption Percentile	0.18^{***}	0.19^{***}	0.12^{**}
	(0.06)	(0.06)	(0.06)
Cultivated Area	0.77^{***}	0.77^{***}	0.77^{***}
	(0.04)	(0.04)	(0.04)
Awareness about rice MSP	−0.01	−0.01	−0.01
	(0.02)	(0.02)	(0.02)
Awareness about wheat MSP	0.12^{***}	0.12^{***}	0.12^{***}
	(0.02)	(0.02)	(0.02)
Land Owned	−0.01	−0.01	−0.01
	(0.01)	(0.01)	(0.01)
Household Size	0.01^{***}	0.01^{**}	0.01^{*}
	(0.003)	(0.003)	(0.003)
Middle school or higher education	−0.01	−0.01	−0.01
	(0.01)	(0.01)	(0.01)
Received agricultural training	−0.07	−0.07	−0.07
	(0.04)	(0.04)	(0.04)
Non-farm expenses	−0.0000	−0.0000	−0.0000
	(0.0000)	(0.0000)	(0.0000)
Non-farm value	0.0000	0.0000	0.0000
	(0.0000)	(0.0000)	(0.0000)
Cumulative monthly rain	−0.0000	−0.0000	−0.0000
	(0.0000)	(0.0000)	(0.0000)
Constant	-0.25^{***}	-0.26^{***}	-0.20^{***}
	(0.07)	(0.07)	(0.07)
State Controls?	Yes	Yes	Yes
Observations	11,182	11,182	11,182
Adjusted R^2	0.84	0.84	0.84

Note: *p<0.1; **p<0.05; ***p<0.01

Standard errors are clustered at the district level.

Linear regression of groundwater irrigated area during dry season (*Rabi*) on consumption percentiles of households estimated using equation 2 and nationally representative survey data of agricultural households in 2012-13, see Data. Policy Change PC is a binary indicator for districts that were majorly electrified after policy change (methods). Consumption percentiles are constructed using all agricultural households across both PC and non-PC districts. The lowest consumption percentile (15th) in non-Policy Change (non-PC) district has been left out. † Specification#2 excludes free collection and specification#3 excludes free collection, gifts and loans and own produce.

Supplementary Table 8: Groundwater irrigation group means in Madhya Pradesh during 2013

	non-PC districts	PC districts	p-value
Wells with electric pumps (#)	73,130.25	40,407.31	0.08
Wheat produced during Rabi (tons)	419,748.87	222,572.85	0.02
Rabi irrigated area (ha)	180,635.50	94,657.66	0.01
Cumulative average *Rabi* rain (mm)	45.60	52.72	0.54
Kharif irrigated area (ha)	43,466.88	44,305.93	0.97
Cumulative average *Kharif* rain (mm)	1,427.60	1,253.08	0.02

Two-sided t-tests between Policy Change (PC) and non-Policy Change (non-PC) districts in Madhya Pradesh using groundwater irrigation data is sourced from panel data (n=45). Wheat production data is averaged over 2011-12 to 2015-16 and is sourced from Directorate of Economics and Statistics, Ministry Of Agriculture and Farmers Welfare.

References

Asher, S., Lunt, T., Matsuura, R., and Novosad, P. (2021). Development Research at High Geographic Resolution: An Analysis of Night Lights, Firms, and Poverty in India using the SHRUG Open Data Platform. *The World Bank Economic Review*.

Census of India, G. o. I. (1991). Population Census of India 1991.

Census of India, G. o. I. (2011). Census of India 2011: Metadata.

Elvidge, C. D., Feng-Chi, H., Baugh, K. E., and Ghosh, T. (2014). National trends on satellite-observed lighting. *Global urban monitoring and assessment through earth observation*, 23:97–118.
