# Supplementary material for: The changing impact of rural electrification on Indian agriculture
Source: Nat Commun. 2023 Oct 25;14:6780. doi: 10.1038/s41467-023-42533-7 (PMC10600167; doi:10.1038/s41467-023-42533-7)
Supplement: Supplementary file 2 — Reporting Summary [file 41467_2023_42533_MOESM2_ESM.pdf]

nature portfolio

Corresponding author(s): Sudatta Ray

Last updated by author(s): Oct 11, 2023

Reporting Summary

Nature Portfolio wishes to improve the reproducibility of the work that we publish. This form provides structure for consistency and transparency in reporting. For further information on Nature Portfolio policies, see our Editorial Policies and the Editorial Policy Checklist.

Statistics

For all statistical analyses, confirm that the following items are present in the figure legend, table legend, main text, or Methods section.

n/a	Confirmed	
☐	☒	The exact sample size (n) for each experimental group/condition, given as a discrete number and unit of measurement
☒	☐	A statement on whether measurements were taken from distinct samples or whether the same sample was measured repeatedly
☐	☒	The statistical test(s) used AND whether they are one- or two-sided<br>*Only common tests should be described solely by name; describe more complex techniques in the Methods section.*
☐	☒	A description of all covariates tested
☐	☒	A description of any assumptions or corrections, such as tests of normality and adjustment for multiple comparisons
☐	☒	A full description of the statistical parameters including central tendency (e.g. means) or other basic estimates (e.g. regression coefficient) AND variation (e.g. standard deviation) or associated estimates of uncertainty (e.g. confidence intervals)
☐	☒	For null hypothesis testing, the test statistic (e.g. F, t, r) with confidence intervals, effect sizes, degrees of freedom and P value noted<br>*Give P values as exact values whenever suitable.*
☒	☐	For Bayesian analysis, information on the choice of priors and Markov chain Monte Carlo settings
☒	☐	For hierarchical and complex designs, identification of the appropriate level for tests and full reporting of outcomes
☒	☐	Estimates of effect sizes (e.g. Cohen's d, Pearson's r), indicating how they were calculated

Our web collection on statistics for biologists contains articles on many of the points above.

Software and code

Policy information about availability of computer code

Data collection	We use government-sourced secondary data for all our analyses. No software was used for any data collection.
Data analysis	All data were processed and analysed in R Studio (Version 2023.09.0+463. We used ggplot2 (Version 3.4.3), maptools (V ersion1.1-8) and pacman (Version 0.5.1) packages to generate plots and maps. Fixest (Version 0.11.1) and Stats (Version 4.2.2) packages in R were used to run the analyses. Installation code for the Stata packages used in the analysis is included in the script files.

For manuscripts utilizing custom algorithms or software that are central to the research but not yet described in published literature, software must be made available to editors and reviewers. We strongly encourage code deposition in a community repository (e.g. GitHub). See the Nature Portfolio guidelines for submitting code & software for further information.

Data

Policy information about availability of data

All manuscripts must include a data availability statement. This statement should provide the following information, where applicable:

- Accession codes, unique identifiers, or web links for publicly available datasets
- A description of any restrictions on data availability
- For clinical datasets or third party data, please ensure that the statement adheres to our policy

All data required for replication are publicly available on Zenodo at https://doi.org/10.5281/zenodo.8430025.

Research involving human participants, their data, or biological material

Policy information about studies with human participants or human data. See also policy information about sex, gender (identity/presentation), and sexual orientation and race, ethnicity and racism.

Reporting on sex and gender	n/a
Reporting on race, ethnicity, or other socially relevant groupings	n/a
Population characteristics	n/a
Recruitment	n/a
Ethics oversight	n/a

Note that full information on the approval of the study protocol must also be provided in the manuscript.

Field-specific reporting

Please select the one below that is the best fit for your research. If you are not sure, read the appropriate sections before making your selection.

☐ Life sciences ☒ Behavioural & social sciences ☐ Ecological, evolutionary & environmental sciences

For a reference copy of the document with all sections, see nature.com/documents/nr-reporting-summary-flat.pdf

Behavioural & social sciences study design

All studies must disclose on these points even when the disclosure is negative.

Study description	We estimate the relationship between change in rural electrified households and the change in electrified groundwater wells between 1986 to 2013 across 323 districts in India. We match various government sourced datasets to create a district-level panel data of total and electrified households, total and electrified groundwater wells, area irrigated by season and long-run average rainfall. We also use sample data of agricultural households collected by the Government of India, National Sample Survey Organization for our secondary analyses on irrigation outcomes at the household-level. All data compiled are quantitative.
Research sample	We estimate the relationship between change in rural electrified households and the change in electrified groundwater wells between 1986 to 2013 across 323 districts in India. We match various government sourced datasets to create a district-level panel data of total and electrified households, total and electrified groundwater wells, area irrigated by season and long-run average rainfall. We also use sample data of agricultural households collected by the Government of India, National Sample Survey Organization for our secondary analyses on irrigation outcomes at the household-level. All data compiled are quantitative.
Sampling strategy	We estimate the relationship between change in rural electrified households and the change in electrified groundwater wells between 1986 to 2013 across 323 districts in India. We match various government sourced datasets to create a district-level panel data of total and electrified households, total and electrified groundwater wells, area irrigated by season and long-run average rainfall. We also use sample data of agricultural households collected by the Government of India, National Sample Survey Organization for our secondary analyses on irrigation outcomes at the household-level. All data compiled are quantitative.
Data collection	All data required for replication are publicly available on Zenodo at https://doi.org/10.5281/zenodo.8430025.<br><br>We use government-sourced secondary data for all our analyses. Data are publicly available from government websites -<br>1.1991, 2001 and 2011 rounds of population and household census of India from https://censusindia.gov.in/census.website/data/census-tables<br>2.First, third and fifth rounds of the Minor Irrigation Census from http://164.100.229.38/state-wise-reports<br>3. Situation Assessment Survey of Agricultural Households, 2013 by the National Sample Survey Organization - http://microdata.gov.in/nada43/index.php/catalog/133<br>4. Rainfall data from the Indian Meteorological Department<br>5. Groundwater Data by the Central Groundwater Board from https://indiawris.gov.in/wris/<br>6. District-level parameters including area cultivated, irrigated and water district by the International crops Research Onstitute for the Semi-arid Tropics and the Tata Cornell Institute (ICRISAT-TCL) from http://data.icrisat.org/dld/src/crops.html
Timing	We use three waves of population census data for 1991, 2001 and 2011. We also use three waves of minor irrigation census data for the years 1986, 2000 and 2013.
Data exclusions	Districts which were missing minor irrigation census data in any of the rounds were dropped. We also dropped districts whose boundaries could not be consistently matched.
Non-participation	This study used government sourced secondary data. There were no participants involved in the study

Randomization	We use the full set of available census data, no randomization was therefore required.

Reporting for specific materials, systems and methods

We require information from authors about some types of materials, experimental systems and methods used in many studies. Here, indicate whether each material, system or method listed is relevant to your study. If you are not sure if a list item applies to your research, read the appropriate section before selecting a response.

Materials & experimental systems

n/a	Involved in the study
☒	☐ Antibodies
☒	☐ Eukaryotic cell lines
☒	☐ Palaeontology and archaeology
☒	☐ Animals and other organisms
☒	☐ Clinical data
☒	☐ Dual use research of concern
☒	☐ Plants

Methods

n/a	Involved in the study
☒	☐ ChIP-seq
☒	☐ Flow cytometry
☒	☐ MRI-based neuroimaging

Plants

Seed stocks	*Report on the source of all seed stocks or other plant material used. If applicable, state the seed stock centre and catalogue number. If plant specimens were collected from the field, describe the collection location, date and sampling procedures.*
Novel plant genotypes	*Describe the methods by which all novel plant genotypes were produced. This includes those generated by transgenic approaches, gene editing, chemical/radiation-based mutagenesis and hybridization. For transgenic lines, describe the transformation method, the number of independent lines analyzed and the generation upon which experiments were performed. For gene-edited lines, describe the editor used, the endogenous sequence targeted for editing, the targeting guide RNA sequence (if applicable) and how the editor was applied.*
Authentication	*Describe any authentication procedures for each seed stock used or novel genotype generated. Describe any experiments used to assess the effect of a mutation and, where applicable, how potential secondary effects (e.g. second site T-DNA insertions, mosiacism, off-target gene editing) were examined.*
